# Compact Optical Fiber 3D Shape Sensor Based on a Pair of Orthogonal Tilted Fiber Bragg Gratings

**DOI:** 10.1038/srep17415

**Published:** 2015-11-30

**Authors:** Dingyi Feng, Wenjun Zhou, Xueguang Qiao, Jacques Albert

**Affiliations:** 1 Department of Physics, Northwest University, Xi’an, 710069, China; 2 Department of Electronics, Carleton University, 1125 Colonel by Drive, Ottawa, Ontario K1S 5B6, Canada

## Abstract

In this work, a compact fiber-optic 3D shape sensor consisting of two serially connected 2° tilted fiber Bragg gratings (TFBGs) is proposed, where the orientations of the grating planes of the two TFBGs are orthogonal. The measurement of the reflective transmission spectrum from the pair of TFBGs was implemented by Fresnel reflection of the cleaved fiber end. The two groups of cladding mode resonances in the reflection spectrum respond differentially to bending, which allows for the unique determination of the magnitude and orientation of the bend plane (i.e. with a ± 180 degree uncertainty). Bending responses ranging from −0.33 to + 0.21 dB/m^−1^ (depending on orientation) are experimentally demonstrated with bending from 0 to 3.03 m^−1^. In the third (axial) direction, the strain is obtained directly by the shift of the TFBG Bragg wavelengths with a sensitivity of 1.06 pm/με.

Fiber Bragg grating (FBG) based sensors have been widely used for static/dynamic structural deformation in various engineering applications including oil well exploration, pipeline monitoring and maintaining and structural health monitoring^1^. In order for FBGs to measure shape and 3D deformations in general they are generally used in orthogonally arranged arrays where each FBG measures one component of the 3D strain as a wavelength shift of its reflection spectrum peak^2^,^3^,^4^,^5^,^6^,^7^,^8^,^9^. Because of the additional mechanical transducing methods required, these sensors tend to occupy relatively large volumes and to require relatively complex assembly that are difficult to make resistant to damage. To avoid these problems, it has recently been demonstrated that 3D shape sensing could be realized with series of FBGs embedded off axis along different directions inside optical fibers^10^,^11^. In all these cases, a sequence of FBGs with different periods are fabricated and interrogated by multichannel spectral monitoring systems. These compact, all-fiber shape sensors have been optimized for large curvatures (defined as the inverse of the bending radius *R*) typically greater than 10 m^−1^ and they require advanced fabrication techniques using femtosecond laser systems to locate FBGs at precise locations in the cross section of optical fibers. A much simpler variant of this had been proposed earlier in which three fibers packaged parallel to each other in a triangular arrangement of their axes, and each containing a FBG, were used to measure curvature in the range of 0 to 10 m^−1^, plane of curvature, and temperature simultaneously^12^. In the approach to be presented here, the azimuthal symmetry breaking properties of tilted FBGs (TFBGs) will be used to achieve similar measurements with a single grating^13^,^14^,^15^,^16^. In a weakly tilted TFBG the grating couples light from the core to the cladding at a large number of wavelengths (depending on tilt angle and grating period) and the amount of coupling depends on the overlap between the modes involved and the tilted grating planes. It is also possible to concatenate TFBGs at different periods, tilt angles and tilt plane orientation, thus creating opportunities for multi-parameter sensing^17^.

We propose and demonstrate a novel approach for 3D shape sensing by using two weakly titled Bragg gratings spliced together such that their tilt plane directions are oriented ~90 degree from each other. The two gratings are followed by a cleaved end face to reflect the transmission signal back into the input fiber by Fresnel reflection. The tilt of the grating planes is fixed at 2 degrees in order for the total spectral widths of the cladding mode resonance spectra remain sufficiently small (about 20 nm)^18^, such that wavelength demultiplexing of the responses of the two gratings is possible within a reasonable spectral window. In a packaged device, a mirror can be coated on the end cleave to increase the signal coming back to the interrogation system but this is not essential. A similar structure with a serial arrangement of TFBGs oriented along different tilt planes has been demonstrated earlier for the purpose of polarization-resolved monitoring of the power carried by optical fibers in telecommunication systems^19^. It will be shown experimentally that this simple structure allows the measurement of the magnitude and direction of relatively small curvatures between 0 and 3 m^−1^, as well as the determination of the strain along the fiber axis, thus completing the requirement for a full 3D shape sensor. These results are explained by the fact that the coupling to certain cladding modes increases for bending along the tilt plane but decreases for bending in the orthogonal direction^20^. For the axial component of the strain measurement, the wavelength shift of the core mode resonance of either of the two gratings is used. The proposed sensing configuration is monolithic and compact, made up only of a piece of optical fiber less than 4 cm long. Similarly to conventional FBG strain gauges, this 3D shape sensing device can be embedded into structures and laminates for robustness.

## Results and Discussion

**Figure 1:**
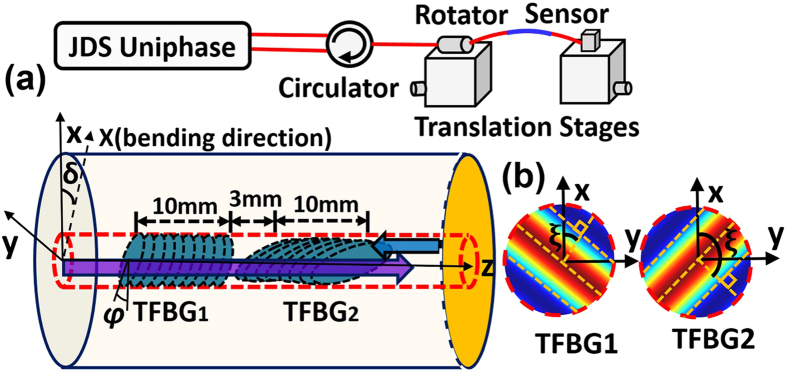
Schematic diagrams of experimental setup for bending and strain measurements (a) and the pair of orthogonal TFBGs (b). The relative orientations of the grating planes of the two TFBGs are shown in the x-y plane.

**Figure 2:**
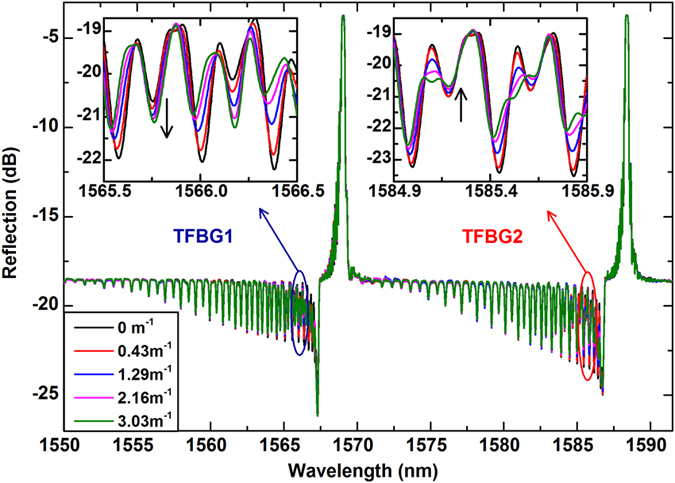
Transmission spectra of the dual orthogonal TFBGs for various curvatures. Insets show the bending-induced spectral responses of the cladding mode resonances around 1565.8 nm (TFBG1) and 1585.2 nm (TFBG2), respectively.

The proposed sensing system is shown in Fig. 1. Here we used two TFBGs with a tilt angle φ of 2 degree connected in series with their tilt orientation at ~90 degrees from each other, a followed by a cleaved end face acting as a mirror. The 2D bending in the plane perpendicular to the fiber axis is first described. A tilt direction ξ relative to the horizontal axis of the transverse coordinate system and a spatial bending direction δ are defined respectively for orienting the device in three dimensional space, as shown in Fig. 1(b). Each grating has a length of 10 mm. The distance between TFBG1and TFBG2 is 3 mm. The reflected spectrum of the fabricated sensing structure is shown in Fig. 2 for several curvature values from 0 to 3.03 m^−1^. The large positive peaks correspond to the core mode coupling (Bragg peak) from the two wavelength-offset gratings and they remain unaffected by bending, as expected. Narrowband dips in the spectrum correspond to light extracted from the core and coupled to individual cladding modes. For each of the gratings a zoomed inset shows a spectral region located approximately 3 nm away from the Bragg peak where clear measurable changes can be observed upon bending. For each of the gratings a zoomed inset shows a spectral region located approximately 3 nm away from the Bragg peak where clear measurable changes can be observed upon bending. In particular, there is a resonance located exactly 3.2 nm away from each Bragg peak that is seen to increase in amplitude (near 1565.8 nm) for one grating TFBG1 and to decrease (near 1585.2 nm) for the other grating TFBG2. The difference is due to the angle between the direction of bending and the tilt plane of the gratings. It is important to point out that the most directionally sensitive resonance in each spectrum (i.e. in each TFBG) is unambiguously identifiable by its wavelength relative to the Bragg peak wavelength. The phase matching condition between the core mode and each cladding mode imposes a one to one relationship between the resonance position and the cladding mode to which the grating couples the incident core mode guided light. For a TFBG with a tilt angle φ, the standard phase matching condition can be expressed by the wavelength (*λ*_*i*_) where the resonance is observed as follows^18^:


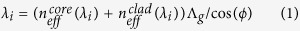


Where 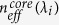 and 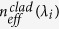 are the effective indices of the core mode and cladding mode at the wavelengths of *λ*_*i*_, respectively, and Λ_g_ is the period of the interference pattern that is used create the grating.

This cladding mode as a well-defined effective index of 1.4417 and can be identified in any TFBG spectrum from a simple calculation involving the grating period and tilt angle. Therefore, the resonance chosen, exactly 3.2 nm from the Bragg peak, can be found and measured in any other TFBG. Furthermore, the amplitude changes of this resonance can be referenced to the power transmission level of the Bragg peak, which is insensitive to both bending and axial strain. While other resonances can be seen to be somewhat more sensitive to bending, their sensitivity is weakly dependent on bend direction and therefore cannot be used for 2D bend sensing.

**Figure 3:**
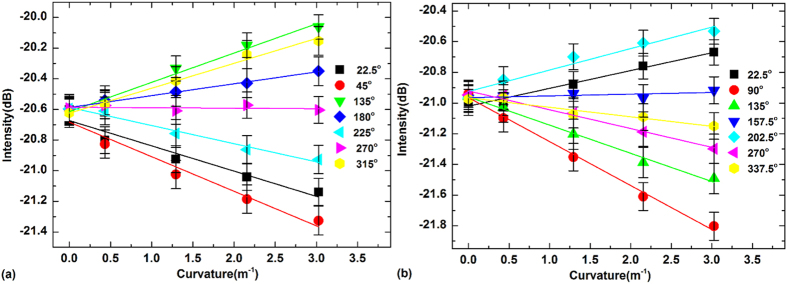
The intensity response of the selected modal resonance to bending of TFBG1 (a) and TFBG2 (b) at bending orientations from 0 to 360^0^, respectively.

The amplitude (largest attenuation dip value in the spectrum, relative to the fixed level of the Bragg peak) of these two cladding modes resonances were measured for various values of the curvature for different bending azimuthal orientations between 0 and 180 degrees around the fiber axis at an interval of 22.5 degrees. Figure 3 shows a typical set of results for TFBG1 (Fig. 3(a)) and TFBG2 (Fig. 3(b)). For each orientation, the amplitude change of the cladding mode resonance demonstrates good linearity from which the bend sensitivity can be calculated. However the individual response of each grating is insufficient to determine the bending magnitude because of the orientation dependence (this problem was noted in the original paper on bend detection using TFBGs)^20^. But here we have two orthogonal responses and it will now be shown that a suitable combination of these two responses provides sufficient information to calculate the bend magnitude and the orientation of the bending plane (but not the absolute direction: there remains an uncertainty of ±180 degrees within the bend plane).

**Figure 4:**
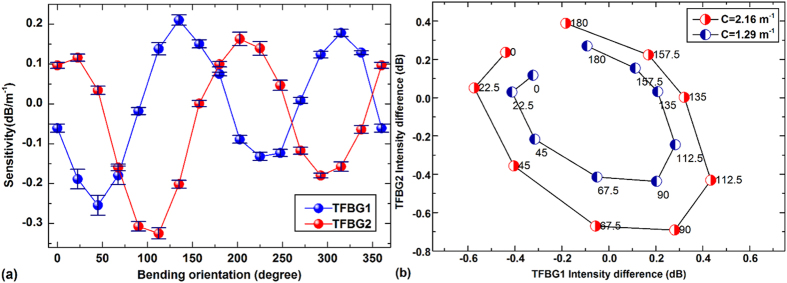
(**a**) Sensitivities of the two selected cladding modes versus bending orientation from 0° to 360°; (**b**) Intensity differences of two TFBGs under the curvatures of 1.29 m^−1^ and 2.16 m^−1^ versus bending orientation.

Figure 4a indicates how the responses from the two TFBGs vary with bend orientation for a given bend magnitude. It is clear that for each TFBG the response is approximately periodical with bend orientation with a period of 180 degrees (consistent with prior findings that the bend sensitivity maximizes in one particular orientation), but also that the two approximate sine-curves have a phase difference of 78 degrees, corresponding to the actual angular difference between the orientations of the tilt planes of the two gratings. The maximum sensitivities are −0.26 dB/m^−1^ and + 0.21 dB/m^−1^ at a measurement wavelength of 1565.75 nm, and −0.33 dB/m^−1^ and + 0.16 dB/m^−1^ at 1585.18 nm, respectively. Also, it can be seen from the curve that the first half plane (0–180 degree) exhibits higher amplitude compared with the second half plane (180–360 degree). This last finding was unexpected and will be discussed below. In order to further emphasize that measurements at these two wavelengths are sufficient to determine the magnitude and orientation of bending, Fig. 4(b) plots the results of Fig. 4(a) in parametric form, i.e. with the change in resonance amplitude of the two gratings relative to their initial value as horizontal and vertical coordinates respectively, and the bend orientation as a parameter labeled on each point (for two values of the bend magnitude). Clearly, each point in the plane of Fig. 4(b) corresponds to a different curvature, orientation and magnitude, as required. The distorted oval shapes of these curves reflects the fact that: a) the two TFBGs were not exactly at 90 degrees to each other; and b) the coupling strengths of the two TFBGs were not exactly equal. While it would be possible to improve on these two factors, it is clearly not necessary, as long as the ellipses observed in Fig. 4b are sufficiently open, meaning that the responses at two orientations differing by 90 degrees do not overlap. This is best understood from as follows: if the responses from TFBG1 and TFBG2 become more in phase as a function of bend orientation in Fig. 4a, the ellipses in Fig. 4b will become narrower along their short axis and converge to a single line for two TFBGs with tilt planes oriented in the same direction. Therefore the requirement for orienting the tilt planes of TFBG1 and TFBG2 at right angles to each other is quite relaxed and does not impose a fabrication complexity and cost beyond the range that is acceptable for the kind of applications where such devices might be used.

All of these results can be understood from the basic coupling mechanism in FBGs, and TFBGs in particular. Using coupled-mode theory under weak coupling approximation, the coupling coefficient between the core mode and a cladding mode can be expressed as follows^21^:


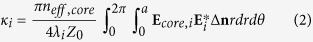


Here, *n*_*eff,core*_ is the effective refractive index in fiber core, *λ*_*i*_ is the wavelength, *Z*_*0*_ *=* 377 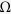, is the free space impedance, ***E***_***core,i***_and ***E***_***i***_are the transverse electric fields of the incident core mode and reflected cladding mode, respectively. For a grating tilted by *φ* degrees relative to the z axis and a tilt direction specified by angle 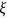 relative to the horizontal axis of the transverse coordinate system (see Fig. 1), the index perturbation is given b: 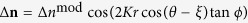, where 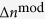 is the amplitude of the index modulation, 2 *K* = 2*π/*Λ is the grating wave-vector corresponding to the projection of the tilted grating period Λ_g_ on the fiber axis (Λ = Λ_g_/cosφ). It is this dependence of the coupling coefficient on azimuthal angle which makes it sensitive to bend orientation. The radial integration is performed from 0 to the core radius *a*, because the photosensitivity of the conventional gratings that we use is limited to the core region, making Δn = 0 elsewhere. When the fiber is bent, an additional perturbation of the refractive index profile occurs across the fiber cross section and this has two effects: the mode fields shift laterally along the bend plane direction (defined here as angle δ relative to the x axis in the 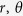 plane) and an additional term must be included in the refractive index perturbation used in the coupling integral (Eqn. 2). As mentioned in^21^ for standard FBGs, when the fiber is bent with a curvature *C* in direction δ, the refractive index perturbation 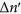 can be written as 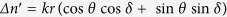, where *k* is equal to 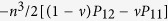, with *n* the refractive i*n*dex, *v* the Poisson ratio, and *P*_*11*_ and *P*_*12*_ are the photoelastic constants of silica glass.

As a result, the modified coupling coefficient takes the following form:


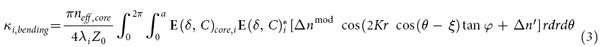



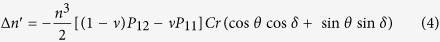


The dependence of the mode fields on the orientation and magnitude of the bend (δ, C) has been explicitly indicated in Eqn. 3. Since the electric field of the core mode is symmetric with respect to the center of the fiber, the effect of bending will be different for modes that are either symmetric or anti-symmetric. In some case the coupling will increase and in some cases it will decrease. The full evaluation of Eqn. 3 will require extensive numerical calculations and will be reported elsewhere. For the purpose of explaining the experimental results obtained here however, the theory presented indicates that since the sign of the coupling coefficient has no effect on the magnitude of the coupled power between the core and a given cladding mode, the coupling strength as expressed by Eqn. 3 should be a periodic function of δ, with a period of 180 degrees. Furthermore, the maximum coupling change must depend on the difference between the bend direction *δ* and the tilt plane direction 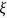. It remains to explain the small residual asymmetry between the 0–180 degree bend results and those along the 180–360 degrees, as well as the fact that the response at 0 degrees is not exactly equal to that at 180 degrees. A possible but unverified explanation comes from the fact that our experimental method to write TFBGs (excimer laser exposure of hydrogen-loaded fibers) produces a small asymmetry of the UV induced refractive index profile along the beam irradiation direction^22^, which is also the tilt plane direction in our devices. As a result, the as-written TFBG prior to bending would have some mode field offsets and cross-sectional index variations (as if the fiber was already bent slightly). It is worth mentioning that more precise results might be obtained if polarized light interrogation of the TFBG pair was used^16^, because of the dependence of the coupling coefficient on scalar products of the vector fields. However, this would also contribute to system complexity and cost and appears unnecessary.

**Figure 5:**
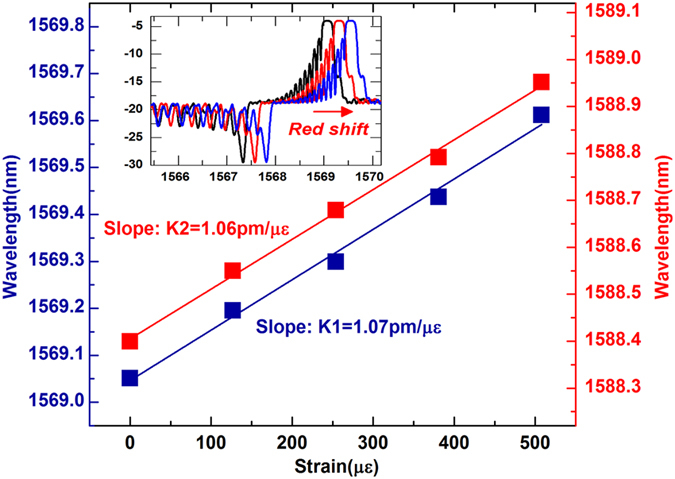
Bragg wavelengths of two TFBGs versus the applied axial strain. Insert indicates reflections of the Bragg peak of TFBG1 under different strains.

**Figure 6:**
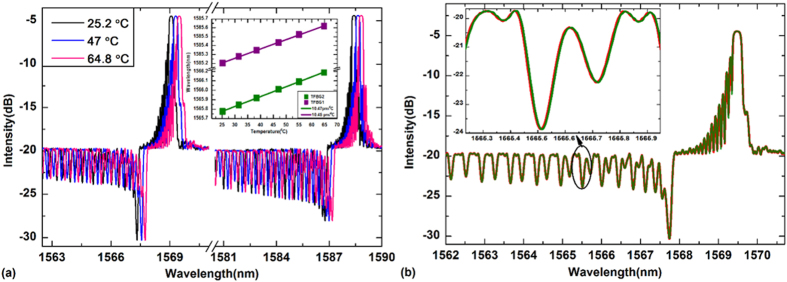
(**a**) Spectra responses under different temperatures. Insert indicates the wavelength shifts of TFBG1 and TFBG2 versus temperature, respectively; (**b**) Overlap of the two spectra for temperatures of 25.2 °C and 64.8 °C, using the Bragg peak to determine the amount of shift needed. The insert shows perfect overlap between the resonances used to measure curvature (3.2 nm from the Bragg peak).

Lastly, the third direction for full 3D shape sensing is discussed. Similarly with normal FBGs^23^, the wavelength of the core mode resonance of a TFBG depends on two factors: the effective index of the fiber core and the axial grating period Λ. When the sensor is stretched symmetrically along z-axis, both factors are changed, contributing to a shift of the Bragg wavelength. Figure 5 shows the experimental results for the red-shifts of the TFBG1 spectrum (as shown in insert) and the strain sensitives K1 to TFBG1 (blue line) and K2 to TFBG2 (red line), respectively. These wavelength shifts provide a measure of axial strain as long as the temperature of the system does not change: the axial strain sensitivity of these TFBGs suffers from the same cross-sensitivity to temperature as the widely used FBG strain sensors. In order to be able to determine the 3D shape uniquely with this proposed novel device, a separate temperature measurement is required and its effect removed from the measured spectral shifts. As shown in Fig. 6, the spectra show negligible deformation to temperature perturbations (which can be clearly seen by overlapping spectra under different temperatures from 25.2 to 65 °C, as seen in Fig. 6(b)) with the power change within ± 0.01 dB for the resonance 3.2 nm from the Bragg peak, apart from the overall wavelength shift with a sensitivity ~10.5 pm/°C (as shown in insert in Fig. 6(a)). This shows that temperature-induced shifts have no effect on curvature measurements.

A full 3D shape measurement then proceeds as follows: 1) measure the wavelengths of the two Bragg peaks; 2) correct the observed wavelengths by the separate temperature measurement and calculating the axial strain from the remaining wavelength shift; 3) locate the two bend sensitive resonances at the fixed wavelength position relative to the Bragg peak and measure their power level relative to that of the Bragg peak; 4) use a look-up table extracted from a calibration similar to the results of Fig. 4b (but at more curvature values) to determine the direction and magnitude of the curvature.

## Conclusion

In conclusion, a novel 3D shape sensor based on a pair of spectrally separated orthogonal tilted fiber Bragg gratings followed by a flat cleaved end face was demonstrated. The transmission spectrum of this device has two resonances that respond differentially to bending along perpendicular directions, one for each of the TFBGs. Results demonstrate that bending directions from 0 to 180 degrees and curvature magnitudes between 0 and 3 m^−1^ can be extracted from each pair of resonance transmission values in a single measurement using unpolarized light. The measurement sensitivities obtained from the two TFBGs range from −0.33 to + 0.21 dB/m^−1^, depending on orientation. For the third dimension in shape measurement, the axial strain was obtained from the wavelength of the Bragg peak similarly as for FBGs, with a sensitivity of 1.06 pm/με. Finally, since the bending magnitude and direction are solely based on power measurements at two select wavelengths, high speed interrogation can be used with a bandpass-filtered broadband source or a pair of lasers to detect vibrations or for seismic sensing (applications for which the third dimension is not needed).

## Methods

The TFBGs we used were both written in CORNING SMF 28 fiber using ArF excimer laser light at 193 nm and a phase mask to generate the grating pattern. Each grating is written separately and then one end is cleaved near the grating end. The Bragg peaks of grating pair are selected with the wavelength around 1569.1 nm and 1588.4 nm, respectively. The two gratings are spliced together in a conventional fusion splicer after rotating the tilt plane of one of the gratings relative to the other. The grating plane orientation of a fabricated grating is located by launching visible light in the fiber core and finding in which direction the maximum outcoupling occurs^24^,^25^. In order to have a single ended miniature device, the double grating structure is cleaved downstream from the second grating thereby providing a 4% broadband reflection of the light transmitted through the gratings. Of course, a more suitable broadband end mirror (either a coated mirror or in fiber chirped FBG) would be used in real application to allow embedding the device into a material for instance. A set of standard translation stages with a rotator and a holder is used to apply bends with controlled radii from all possible azimuthal directions, as shown in Fig. 1(a). In order to interrogate the device, non-polarized light is injected to the gratings by a tunable laser source with wavelength range from 1520 to 1630 nm, and the spectrum of the sensor is detected by a JDS Uniphase SWS-OMNI-2 system with a resolution of 0.002 nm.

## Additional Information

**How to cite this article**: Feng, D. *et al.* Compact Optical Fiber 3D Shape Sensor Based on a Pair of Orthogonal Tilted Fiber Bragg Gratings. *Sci. Rep.* **5**, 17415; doi: 10.1038/srep17415 (2015).
